# *Pseudomonas aeruginosa* Overrides the Virulence Inducing Effect of Opioids When It Senses an Abundance of Phosphate

**DOI:** 10.1371/journal.pone.0034883

**Published:** 2012-04-13

**Authors:** Alexander Zaborin, Svetlana Gerdes, Christopher Holbrook, Donald C. Liu, Olga Y. Zaborina, John C. Alverdy

**Affiliations:** 1 University of Chicago Medical Center, Department of Surgery, Chicago, Illinois, United States of America; 2 Fellowship for Interpretation of Genomes, Burr Ridge, Illinois, United States of America; University of Hyderabad, India

## Abstract

The gut during critical illness represents a complex ecology dominated by the presence of healthcare associated pathogens, nutrient scarce conditions, and compensatory host stress signals. We have previously identified key environmental cues, opioids and phosphate depletion that independently activate the virulence of *Pseudomonas aeruginosa*. Opioids induce quinolone signal production (PQS), whereas phosphate depletion leads to a triangulated response between MvfR-PQS, pyoverdin, and phosphosensory/phosphoregulatory systems (PstS-PhoB). Yet how *P. aeruginosa* manages its response to opioids during nutrient scarce conditions when growth is limited and a quorum is unlikely to be achieved is important in the context of pathogenesis in gut during stress. To mimic this environment, we created nutrient poor conditions and exposed *P. aeruginosa* PAO1 to the specific k-opioid receptor agonist U-50,488. Bacterial cells exposed to the k-opioid expressed a striking increase in virulence- and multi-drug resistance-related genes that correlated to a lethal phenotype in *C. elegans* killing assays. Under these conditions, HHQ, a precursor of PQS, rather than PQS itself, became the main inducer for *pqsABCDE* operon expression. *P. aeruginosa* virulence expression in response to k-opioids required PqsE since ΔPqsE was attenuated in its ability to activate virulence- and efflux pumps-related genes. Extracellular inorganic phosphate completely changed the transcriptional response of PAO1 to the k- opioid preventing *pqsABCDE* expression, the activation of multiple virulence- and efflux pumps-related genes, and the ability of *P. aeruginosa* to kill *C. elegans*. These results indicate that when *P. aeruginosa* senses resource abundance in the form of phosphate, it overrides its response to compensatory host signals such as opioids to express a virulent and lethal phenotype. These studies confirm a central role for phosphate in *P. aeruginosa* virulence that might be exploited to design novel anti- virulence strategies.

## Introduction

During chronic illness, immunodeficiency, and exposure to extreme medical interventions such as organ transplantation and chemotherapy, the intestinal tract becomes invariably colonized by multi-drug resistant healthcare associated pathogens (HAP) and serves as the primary source of subsequent infection- related morbidity and mortality [1]–[3]. Much remains to be understood about the local conditions that drive intestinal pathogens to remain as dormant colonizers one moment and invasive and virulent pathogens the next. During critical illness the intestinal tract becomes a rich source of compensatory host signals that can be gathered, processed, and transduced by its colonizing microbiota. Much of this response develops in response to the ability of modern medicine to sustain life through extreme episodes of hypoxia, shock, and inanition. Prolonged use of mechanical support of the heart and lungs, provision of nutrients directly into the bloodstream, and sustained doses of opioids and antibiotics, create a harsh intestinal microenvironment characterized by hypoxia, replacement of the commensal microbiota by hospital associated pathogens, and nutrient depletion. Our previous work in this area demonstrates that many host derived compensatory signals released into the gut under these circumstances can directly signal the quorum sensing circuitry of *P. aeruginosa*. For example, we have previously demonstrated that *P. aeruginosa* virulence is activated by soluble compounds such as opioids [4], [5], nucleosides [6], and cytokines [7] as well as by changes in physico-chemical cues such as pH [8], iron, and phosphate levels [9], [10]. Yet a major question to be addressed is how *P. aeruginosa* responds to compensatory host factors when nutrients are limited and bacterial cell densities remain low as might be encountered in the gut during extreme physiologic stress and its treatment. As we previously identified phosphate to play a pivotal and central role in the process by which *P. aeruginosa* expresses virulence in response to host stress [8], [9], we specifically designed studies to determine if inorganic phosphate supplementation would override the response of *P. aeruginosa* to opioids. Results highlight potential mechanisms by which *P. aeruginosa* is capable of co-processing multiple input signals such that it can override virulence expression when it senses the abundance of key nutrients such as phosphate. The context under which this response may have evolved has far reaching implications and could lead to novel preventative anti- virulence strategies using phosphate-based approach.

## Results

### In a nutrient depleted environment, *P. aeruginosa* can adopt either a “harmless” (energy conserving) or “virulent” (energy consuming) phenotype depending on the absence or presence of k-opioid

**Figure 1:**
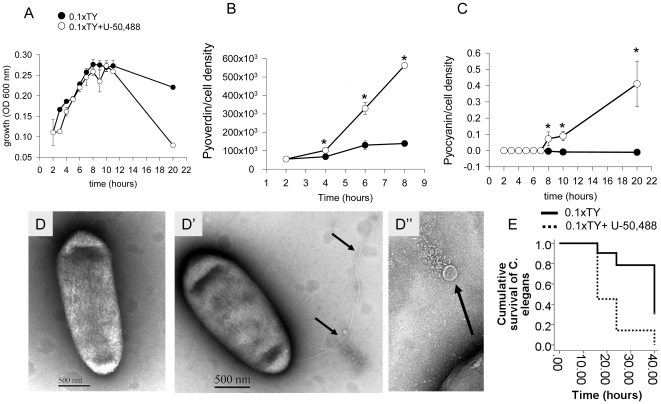
*P. aeruginosa* PAO1 response to k-opioid during growth in nutrient poor medium. (**A**) Effect of 200 µM U-50,488 on PAO1 cell density, n = 3. (**B**) Pyoverdin production normalized to cell density, n = 3, *p<0.01. (**C**) Pyocyanin production normalized to cell density, n = 3, *p<0.05. (**D–D″**) Transmission electron microscopy images of PAO1 cells grown in (**D**) 0.1xTY, and (**D′, D″**) grown in 0.1xTY supplemented with 200 µM U-50,488. Flagella (**D′**) and vesicles (**D″**) are shown by arrows. (**E**) Kaplan-Meier survival curves in *C. elegans* feeding in *P. aeruginosa* PAO1 grown in poor nutrient medium vs poor nutrient medium containing 50 µM U-50,488. Cell cultures were collected at 7 hrs. Cumulative survival is represented as n = 7/plate, 3 plates/experiment, p<0.01.

In order to characterize the behavior of *P. aeruginosa* in nutrient depleted medium with or without the addition of k-opioids, we first analyzed its growth, production of virulence factors such as pyocyanin and pyoverdin, cell morphology, and its killing ability. In this experiment, *P. aeruginosa* PAO1 was grown in 10 fold diluted TY medium (0.1xTY) spiked with the specific k-opioid receptor agonist U-50,488 (200 µM). Notably, the bacterial growth rate was low under nutrient limited conditions, and cells reached a late exponential phase at 8 hrs at the density of 0.25–0.3 independent of the presence/absence of the opioid (Fig. 1A). However a striking difference in growth was noted at 20 hrs whereby cell density slightly decreased in non- opioid treated *P. aeruginosa* versus near complete cessation of growth when the k-opioid was present (Fig. 1A). A negligible production of pyoverdin and no production of pyocyanin was noted in non- opioid treated *P. aeruginosa* whereas enhanced production of both virulence factors was observed in the presence of k-opioid (Fig. 1B,C). Analysis of cell morphology by transmission electron microscopy in PAO1 collected after 5 hrs of growth revealed normally shaped cells when PAO1 was grown in nutrient poor medium (Fig. 1D). Neither flagella, nor pili were identified (Fig. 1D). Conversely, following 3 hours of exposure of PAO1 to the k- opioid, flagellation and vesicle formation were observed by electron microscopy analysis (Fig. 1D′, D″). The filaments were however disintegrated perhaps owing to insufficient energy sources due to relative lack of nutrients.

We next determined the effect of the k-opioid on the ability of PAO1 to kill *C. elegans*. Previously we demonstrated that the progeny of *C. elegans* are significantly decreased when worms feed on *P. aeruginosa* PAO1 grown as lawns on NGM media exposed to k-opioids [4]; however no worm mortality developed under these conditions [4]. Only when we created nutrient poor conditions, as outlined in the current study, did worms die. Therefore, in the present study, worms were pre-starved for 3 hrs on non-seeded plain agarized plates covered with 1 ml of 100 µg/ml kanamycin solution. Pre-starved L4-adult worms were transferred in ø30 mm dishes containing 1.5 ml of PAO1 cultures collected after 7 hrs of growth in 0.1xTY medium that included 5 hrs exposure to the k-opioid U-50,488. Experiments were performed in liquid media to maintain low bacterial cell densities. Incubation of worms in PAO1 culture was performed at RT and shaking at 40 rpm. Under these nutrient poor conditions, we observed fast killing of *C. elegans* feeding on *P. aeruginosa* PAO1 that reached 60% mortality at 48 hrs (Fig. 1E); an effect that was accentuated during exposure to opioids. Dose response experiments demonstrated this accentuated response (>80% mortality of *C. elegans* at 24 hrs) at opioid concentrations as low as 50 µM (Fig. 1E). No mortality was observed at 24 hrs when worms were incubated with OP50 and there was a 10% mortality with OP50 exposed to U-50,488 (n = 7/plate, 3 plates/experiment) suggesting that the shift of PAO1 to a virulent phenotype but not U-50,488 itself caused fast killing in worms. We next hypothesized that the enhanced killing ability of *P. aeruginosa* would allow it to acquire host nutrient resources and thus maintain its population level. We therefore counted *P. aeruginosa* cells inside the worm dishes after overnight incubation with *C. elegans* by plating 10 fold dilutions on Pseudomonas isolation agar (PIA). Counts demonstrated 4–7×10^7^ CFU/ml in 0.1xTY and 2–4×10^8^ colonies in 0.1xTY+U-50,488 (corresponds to ∼0.3 OD 600 nm) suggesting that *P. aeruginosa* populations remain stable if not increased in poor nutrient media spiked with opioids. These data suggest that under conditions of limited nutrients, virulence expression imposes a cost tradeoff that may result in either overall bacterial population loss or host nutrient acquisition.

### Transcriptional response of PAO1 to the k-opioid in nutrient poor medium

In order to determine the mechanisms by which *P. aeruginosa* produces quorum sensing-related virulence production at low cell density during growth in nutrient poor media, a genome wide transcriptional analysis was performed in bacterial cells collected after 5 hrs of exposure to k-opioid using Affymetrix microarray chips. Microarray data were deposited in Gene Expression Omnibus (GEO) database, accession number GSE29946, and selected data displayed in Table 1. Analysis of the transcriptional response of the central core of quorum sensing genes revealed enhanced expression of *pqsE* (>2.5 fold) while all other components of core quorum sensing regulation were either downregulated (>2 fold, *lasR*, *lasI*) or demonstrated no response (*rhlR*, *rhlI*). The upregulation of the *pqsABCDE* operon was coupled with down-regulation of the *antABC* operon involved in the degradation of anthranilate that can promote enhanced biosynthesis of quinolone molecules. We also observed a significant increase in the expression of genes involved in phenazine (20–50 fold) and cyanide biosynthesis (15–20 fold). Cyanide has a high affinity for certain sulfur compounds and metallic complexes, particularly those containing cobalt and the trivalent form of iron (Fe^3+^). As such, the cyanide ion can damage S-S bonds, inactivating multiple enzymes, and rapidly combines with iron in cytochrome aa3, interrupting the aerobic respiratory chain. Indeed, respiratory chain gene expression demonstrated a deficiency in the function of electron transport chain in response to the k- opioid as evidenced by the observation that that several cytochrome oxidases were down-regulated including all 3 subunits of cytochrome aa3 oxidase (PA0105–PA0108) and terminal cytochrome oxidase (PA3928–PA3930). Since several cytochrome oxidases were down-regulated, without significant induction of the denitrification chain components, it is likely that bacterial cells used alternative mechanisms of energy generation and maintenance of redox homeostasis. The observation that both phenazine operons were up-regulated may indicate that phenazines and other redox-active secondary metabolites could act as electron acceptors in cellular energy generation and/or in maintenance of the intracellular redox balance as has been proposed by others [11] representing alternative mechanisms of energy generation. We also detected two gene clusters PA3327–PA3336 and PA1211–PA1221 to be significantly induced by the k-opioid. Analysis of predicted polypeptides encoded by these hypothetical operons indicates that each of them controls the synthesis of a secreted secondary metabolite (each gene cluster includes a putative efflux transporter of major facilitator superfamily, and non-ribosomal peptide synthetase modules and related proteins). Significant up-regulation (2.5–15 fold) of multi-drug resistant encoding genes of resistance- nodulation- cell-division (RND) family (PA2528–PA2525, PA4205–PA4208, PA4599–PA4597), outer membrane protein OprG, multidrug efflux transporter PA4990, OprM family Apr (PA1246–PA1249), and putative beta-lactamase PA1797 was observed in response to the k-opioid (Table 1, Table S4) suggesting that exposure to opioids may increase the resistance of *P. aeruginosa* to antibiotics.

**Table 1:** Changes in the expression of selected genes in PAO1 in the response to k-opioid U-50,488 during growth in poor nutrient medium.

	PA ID	Gene name	Fold change, +/− opioid	Product name
**Core QS signaling**	PA1430	*lasR*	−2.2	Transcriptional regulator LasR
	PA1432	*lasI*	−2.37	Autoinducer synthesis protein LasI
	PA3476	*rhlI*	1.03	Autoinducer synthesis protein RhlI
	PA3477	*rhlR*	−1.24	Transcriptional regulator RhlR
	PA1003	*mvfR*	1.028	Transcriptional regulator MvfR
	PA1000	*pqsE*	2.78	Quinolone signal response protein
**Anthranilate degradation**	PA2512	*antA*	−19.58	Anthranilate dioxygenase large subunit
	PA2513	*antB*	−35.73	Anthranilate dioxygenase small subunit
	PA2514	*antC*	−9.87	Anthranilate dioxygenase reductase
**Phenazines**	PA4209	*phzM*	7.87	Probable phenazine-specific methyltransferase
	PA4217	*phzS*	13.02	Flavin-containing monooxygenase
	PA4210/PA1899	*phzA1//phzA2*	26.09	Probable phenazine biosynthesis protein
	PA4211/PA1900	*phzB1//phzB2*	51.04	Probable phenazine biosynthesis protein
	PA4212/PA1901	*phzC1//phzC2*	11.59	Phenazine biosynthesis protein PhzC
	PA4213/PA1902	*phzD1//phzD2*	18.4	Phenazine biosynthesis protein PhzD
	PA4214/PA1903	*phzE1//phzE2*	23.56	Phenazine biosynthesis protein PhzE
	PA4215/PA1904	*phzF1//phzF2*	22.23	Probable phenazine biosynthesis protein
	PA4216/PA1905	*phzG1//phzG2*	18.1	Probable pyridoxamine 5′-phosphate oxidase
**Cyanide**	PA2193	*hcnA*	24.27	Hydrogen cyanide synthase HcnA
	PA2194	*hcnB*	13.03	Hydrogen cyanide synthase HcnB
	PA2195	*hcnC*	14.49	Hydrogen cyanide synthase HcnC
**PA3327–PA3336**	PA3327		4.33	Probable non-ribosomal peptide synthetase
	PA3328		9.4	Probable FAD-dependent monooxygenase
	PA3329		11.91	Hypothetical protein
	PA3330		11.82	Probable short chain dehydrogenase
	PA3331		16.6	Cytochrome P450
	PA3332		14.51	Probable Snoal-like polyketide cyclase
	PA3333	*fabH2*	11.17	3-oxoacyl-[acyl-carrier-protein]synthase III
	PA3334		10.75	Probable acyl carrier protein
	PA3335		4.1	Putative methyltransferase
	PA3336		3.69	Major facilitator superfamily (MFS) transporter
**PA1211−PA1221**	PA1211		2.62	Hypothetical protein, alpha/beta hydrolase fold
	PA1212		5.65	Major facilitator superfamily (MFS) transporter
	PA1213		8.92	Putative monooxygenase/hydrolase
	PA1214		8.74	Putative asparagine synthase
	PA1215		9.22	Acyl-CoA synthetase
	PA1216		25.44	Hypothetical protein
	PA1217		16.02	Probable 2-isopropylmalate synthase
	PA1218		12.37	Putative dioxygenase
	PA1219		4.29	Hypothetical protein
	PA1220		4.49	Hypothetical protein
	PA1221		3.56	Hypothetical protein with AMP-binding domain
**Cytochrome oxidases**	PA0105	*coxB*	−6.06	Cytochrome C oxidase, subunit II
	PA0106	*coxA*	−3.72	Cytochrome C oxidase, subunit I
	PA0107		−2.7	Conserved hypothetical protein
	PA0108	*coIII*	−2.4	Cytochrome C oxidase, subunit III
	PA3928		−3.59	Hypothetical protein
	PA3929	*cioB*	−3.23	Cyanide insensitive terminal oxidase
	PA3930	*cioA*	−3.5	Cyanide insensitive terminal oxidase
**Multi-drug resistance**	PA4205	*mexG*	8.38	Hypothetical protein
	PA4206	*mexH*	4.73	RND efflux membrane fusion protein precursor
	PA4207	*mexI*	2.7	RND efflux transporter
	PA4208	*opmD*	3.39	Probable outer membrane protein precursor
	PA4599	*mexC*	15.2	RND efflux membrane fusion protein precursor
	PA4598	*mexD*	2.98	RND efflux transporter
	PA4597	*oprJ*	1.85	Outer membrane protein precursor
	PA4067	*oprG*	4.85	Outer membrane protein precursor
	PA4990		7.8	SMR multidrug efflux transporter
	PA1246	*aprD*	2.02	Alkaline protease secretion protein
	PA1247	*aprE*	2.68	Alkaline protease secretion protein
	PA1248	*aprF*	2.12	Outer membrane protein precursor
	PA1249	*aprA*	14.66	Alkaline metalloproteinase precursor
	PA1797		10.56	Putative beta-lactamase

### K-opioid synergizes with HHQ to induce the expression of *pqsABCDE* at low cell density

**Figure 2:**
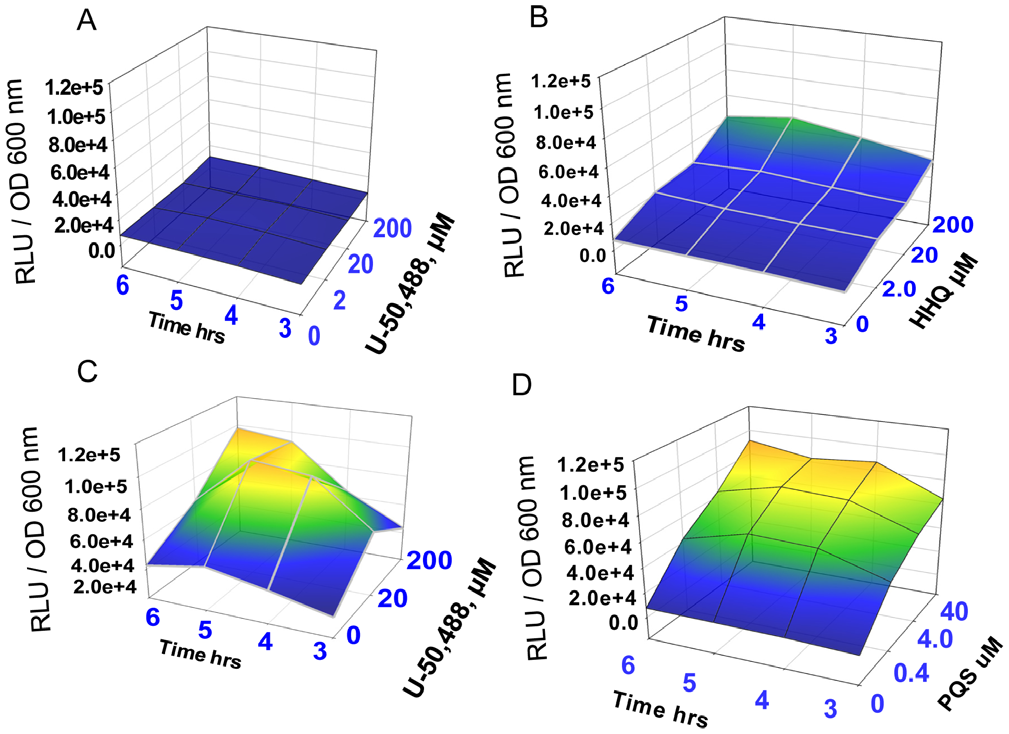
Kappa opioid induces activation of *pqsABCDE* synergistically with HHQ. Luminescence normalized to cell density was measured dynamically in the reporter strain ΔPqsA/*pqsA::luxCDABE* grown in 0.1xTY in the presence of (A) varying doses of U-50,488; (B) varying doses of HHQ; (C) varying doses of U-50,488+200 µM HHQ; and (D) varying doses of PQS.

The first four genes of the *pqsABCDE* (*pqsA–E*)operon are involved in the transformation of anthranilic acid to a series of quinolones including the 2-heptyl-4-quinolone (HHQ), a precursor of quorum sensing molecule PQS (Pseudomonas quinolone signal) [12]–[15]. Further transformation of HHQ to PQS is regulated by the core QS LasRI system [16]. However, down- regulation of *lasR* and *lasI* as well up- regulation of multiple Mex pumps can prevent accumulation of QS autoinducer molecules and thus QS activation [16], [17]. As such, enhanced expression of *pqsA–E* in response to k-opioid was further explored. It is known that PQS is a potent native ligand of the transcriptional regulator MvfR that is required for up-regulation of the *pqsA–E* [18], [19]. It has also been shown that HHQ can activate the transcriptional activity of MvfR, although PQS is 100-fold more potent than HHQ in activating *pqsA–E* transcription [19]. Therefore we examined the activating effect of PQS, HHQ, and U-50,488 on *pqsA–E* transcription in the PAO1 derivative reporter strain ΔPqsA harboring *pqsA::luxCDABE* construct [20]. This reporter strain is deficient in the production of quinolone compounds and therefore luminescence due to expression of the *pqsABCDE* operon can only be induced by exogenous addition of MvfR ligands [21]. We found only a background of luminescence when ΔPqsA/*pqsA::luxCDABE* was exposed to U-50,488 (Fig. 2A), slightly exceeding the baseline level of luminescence induced by 200 µM HHQ (Fig. 2B). However, when we exposed ΔPqsA/*pqsA::luxCDABE* to the 200 µM HHQ combined with U-50,488 at a range of 2–200 µM, we found that U-50,488 enhanced the activating efficiency of HHQ in a concentration dependent manner (Fig. 2C) reaching luminescence on par with PQS (Fig. 2D). These data demonstrate that the synergistic effect of the k-opioid and HHQ is sufficient to upregulate the *pqsABCDE* operon in the absence of PQS.

### PqsE is involved in k-opioid mediated virulence expression in *P. aeruginosa*

**Figure 3:**
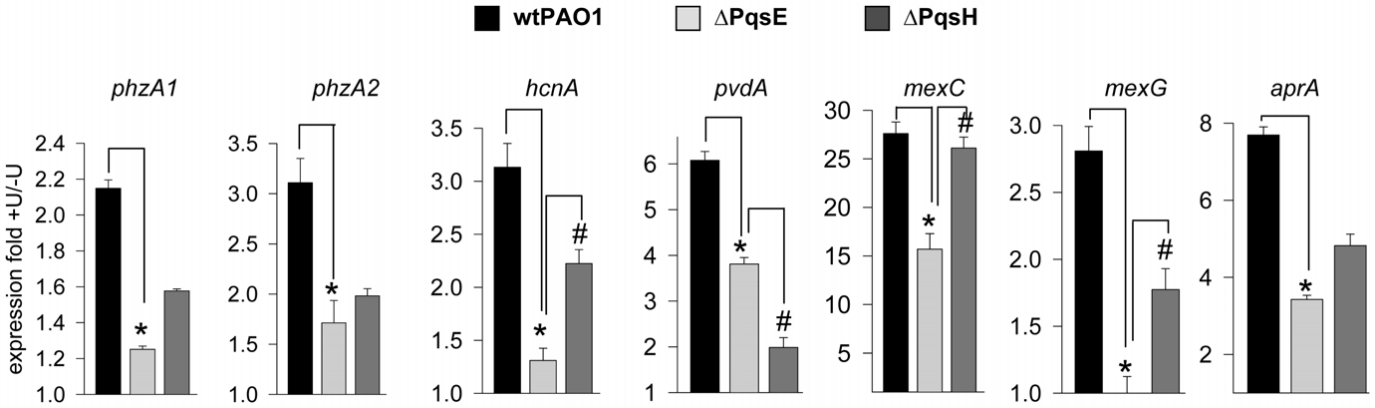
QRT-PCR analysis demonstrating involvement of PqsE and PqsH in the response of *P. aeruginosa* to the k-opioid. Average values with standard deviations are represented. n = 3/variant, *p<0.05 (wtPAO1 vs ΔPqsE); #p<0.05 (ΔPqsE vs ΔPqsH). Results were reproduced in two independent experiments.

The fifth gene of the *pqsABCDE* operon encodes the quinolone signal response protein PqsE which is not required for quinolone biosynthesis but is required for the activation of various downstream virulence genes [22]. We hypothesized that PqsE is involved in the k-opioid mediated virulence effect in *P. aeruginosa*. To demonstrate this we performed qRT-PCR analysis using the ΔPqsE mutant deficient in PqsE [23] and the ΔPqsH mutant deficient in PQS. The isogenic strain NPAO1 (Nottingham collection wt PAO1) was used in these experiments. The expression of genes involved in phenazine (*phzA1*, *phzA2*), pyoverdin (*pvdA*), and cyanide (*hcnA*) biosynthesis, antibiotic resistance (*mexC*, *mexG*, and *aprA*), and the housekeeping gene PA4748 *tpiA* (used for normalization) was measured in *P. aeruginosa* collected at 7 hrs of growth in 0.1xTY and in 0.1xTY supplemented with 200 µM of U-50,488. Data demonstrated attenuated to no transcriptional response to k-opioid in *phzA1*, *hcnA*, *mexC*, *mexG*, and *aprA* in ΔPqsE (Fig. 3) indicating that PqsE represents the major regulatory protein involved in k-opioid- mediated signaling. An attenuated transcriptional response to k-opioid, albeit to a lesser degree (except for *pvdA*), was observed in ΔPqsH confirming our previous findings that the k-opioid can also synergize with PQS [4].

### Extracellular phosphate suppresses the effect of k-opioids on *P. aeruginosa* PAO1 virulence and lethality

**Figure 4:**
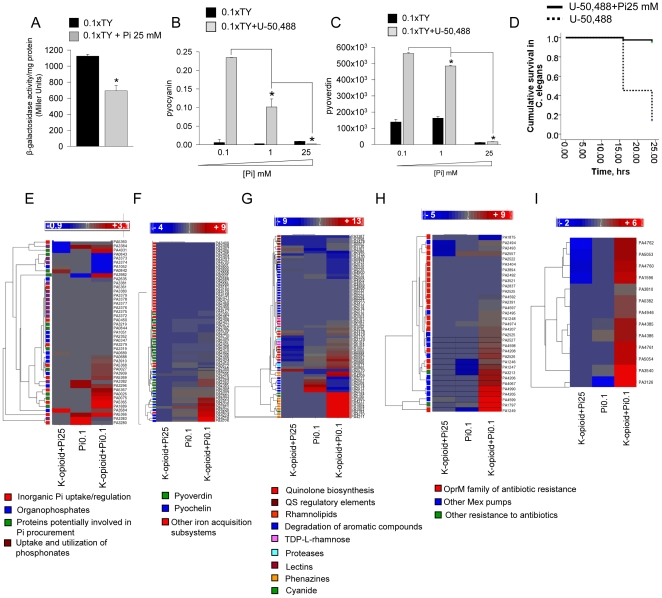
Virulence expression in response to k-opioid at high and low phosphate concentrations. (A) MvfR expression measured by β-galactosidase activity in PAO1/*mvfR'-lacZ*. (B) pyocyanin production normalized to cell density. (C) pyoverdin production normalized to cell density. (D) Mortality in *C. elegans* incubated with PAO1 grown in 0.1xTY media plus 200 µM U-50,488 with and without supplementation with Pi. (E–I) Heat maps of gene expression related to (E) low phosphate signaling/acquisition, (F) low iron signaling/acquisition, (G) quorum sensing, (H) multi-drug resistance, and (I) stress response.

Given that *pqsABCDE* transcription is regulated by MvfR and that extracellular inorganic phosphate concentration is an important regulator of *mvfR* expression [9], we next determined if the addition of phosphate to a nutrient poor medium inhibits the effect of the k-opioid on the virulence/lethal phenotype in *P. aeruginosa* PAO1. First we examined MvfR expression using the reporter *P. aeruginosa* PAO1/*mvfR'::lacZ* [4], [18]. The strain was grown in 0.1xTY or 0.1xTY+25 mM Pi, and β-galactosidase activity was measured at 7 hrs. Results demonstrated that phosphate supplementation significantly attenuated MvfR expression (Fig. 4A). The presence of opioids did not significantly affect MvfR expression under both phosphate concentrations, although there was a trend toward an increase (0.1xTY vs 0.1xTY+U-50,488: 1124.538±21.72 vs 1360.82±235.26; 0.1xTY+Pi25 vs 0.1xTY+Pi25+U-50,488: 680±67.14 vs 832.18±168.75 (Miller Units). n = 3, NS). We also found that phosphate supplementation attenuated opioid-induced pyocyanin (Fig. 4B) and pyoverdin (Fig. 4C) production and mortality in *C. elegans* (Fig. 4D). We next compared transcriptional patterns of *P. aeruginosa* grown in 1.) nutrient poor 0.1xTY, 2.) 0.1xTY+200 µM of U-50,488, and 3.) 0.1xTY+200 µM of U-50,488+Pi 25 mM. For comparison, the fold change for all three groups vs 0.1xTY+Pi25 mM was calculated and heat maps created using Partek analysis (Fig. 4E–I, Tables S1, S2, S3, S4, S5). We specifically focused on the analysis of phosphate signaling/acquisition (Fig. 4E, Table S1), iron signaling and acquisition (Fig. 4F, Table S2, quorum sensing (Fig. 4G, Table S3), multi-drug resistance (Fig. 4H, Table S4), and the stress response (Fig. 4I, Table S5).

Analysis of phosphate signaling/acquisition revealed that *P. aeruginosa* did experience some phosphate limitation in 0.1xTY medium compared to 0.1xTY+Pi25 mM (middle column) as judged by increased expression of *pstB* (PA5366), belonging to low phosphate signaling subsystem; *oprO* (PA3280), the pyrophosphate specific outer membrane porin OprO; alkaline phosphatase encoding *phoA* (PA3296); and genes of phosphonate acquisition subsystem: *phnE* (PA3382), *phnD* (PA3383), and *phnC* (PA3384). Exposure to the k-opioid (right column) led to higher expression of a low phosphate signaling subsystem consisting of *pstS* (PA5369), *pstCAB* operon (PA5368–PA5366), and *phoU* (PA5365) as well as genes involved in Pi procurement such as *ppA* encoding inorganic pyrophosphatase (PA4031), a hypothetical alkaline phosphatase PA1689, and probable phosphoprotein phosphatase *pppA* (PA0075). The k-opioid induced effect on phosphate subsystem can be explained by increased need for phosphate to supply extra energy for synthesis of virulence products. All but *glpD* did not respond to k-opioid when extracellular source of energy (25 mM Pi) was delivered (left column).

Many iron signaling/acquisition genes (Fig. 4F, Table S2) were shown to be activated in 0.1xTY vs 0.1xTY+Pi 25 mM (middle column) including pyoverdin-related *fpvA* (PA2398), *pvdA* (PA2386), *pvdS* (PA2426), and pyochelin-related genes *pchD* (PA4228), *pchC* (PA4229), *pchB* (PA4230), and *pchA* (PA4231). The addition of the k-opioid to 0.1xTY enhanced expression of multiple pyoverdin- (*pvdQ*, *pvdA*, *pvdM*, *pvdN*, *fpvA*) and pyochelin-related genes (*ampP*, *fptB*, *fptA*, *pchG*, *pchF*, *pchE*, *pchD*, *pchA*) but no other iron acquisition related subsystems (right column). The addition of extracellular inorganic phosphate completely suppressed the expression of pyoverdin- and pyochelin related genes (left column). The measurement of pyoverdin production demonstrated a phosphate suppressing effect on iron acquisition systems (Fig. 4C). The effect of phosphate on siderophore-related gene expression and siderophore production [8], [9] as well as the physical interaction between phosphate and iron regulators [24] have been recently reported and current data confirm the important role of phosphate on iron related gene expression.

Analysis of quorum sensing-related gene expression revealed that *pqsABCDE* expression was ∼2 fold higher in 0.1xTY vs 0.1xTY+Pi 25 mM and ∼4 fold higher in the presence of the k-opioid whereas addition of phosphate led to *pqsABCDE* down- regulation. This emphasizes the importance of phosphate as a proximal regulator of MvfR-dependent *pqsABCDE* transcription. The transcription of rhamnolipids-related genes *rhlA* and *rhlB* was induced >2 fold by the k-opioid but this effect was not dependent on phosphate concentration owing to the possibility that that k-opioid mediated signaling might involve alternative undescribed pathways. The expression of genes related to TDP-L-rhamnose, phenazine, cyanide biosynthesis, and proteases *lasA*, *lasB*, and PA3535 was not different between 0.1xTY and 0.1xTY+Pi 25 mM but was markedly induced by the k-opioid (right column). The k-opioid-inducing effect was completely suppressed by phosphate (left column). This was confirmed by the finding that pyocyanin production induced by the k-opioid was suppressed by phosphate in a concentration dependent manner (Fig. 4B). The high level of expression of genes involved in the degradation of aromatic compounds (*catA*, *catC*, PA2511, *antA*, *antB*, *antC*) in 0.1xTY vs 0.1xTY+Pi 25 mM suggests that bacteria attempted to prevent synthesis of precursors of quorum signaling under nutrient poor conditions and perhaps used their aromatic compounds as an amino acid source. During exposure to the k-opioid, the degradation of aromatics was completely abolished which is unexpected when bacteria exist in a nutrient poor environment. The addition of phosphate to the k-opioid in these experiments partially restored the degradation of aromatics suggesting that the opioid was the main factor suppressing the degradation of anthranilate (left column). Interestingly, the expression of *lecA* was induced only in the presence of 25 mM phosphate and k-opioid, again, suggesting that additional pathways are involved in k-opioid processing. Phosphate supplementation almost completely abolished mortality in worms corresponding to down- regulation of multiple virulence genes (Fig. 4D).

Finally the k- opioid induced the expression of genes involved in multi-drug resistance (Fig. 4H, Table S4) and stress response pathways (Fig. 4I, Table S5) (right columns). These genes were completely suppressed in high phosphate medium suggesting that under phosphate sufficient conditions, bacteria remain unstressed and antibiotic resistance is attenuated.

## Discussion

Although it is widely recognized that a growing majority of healthcare acquired pathogens use the intestinal tract as their primary site of colonization, the local conditions that shape bacterial phenotype expression during extreme physiologic stress and its treatment remain unknown. The importance of the intestinal tract reservoir as the major site of antibiotic resistance development and the primary infection source during human critical illness has been emphasized by others [25], [26]. In this report we sought to uncover the molecular mechanisms by which *P. aeruginosa*, one of the most common pathogens to colonize the gut of critically ill humans, co- manages two key but separate local environmental cues that regulate its virulence and lethal effect in the gut, phosphate and opioids. Both phosphate depletion and opioid release appear to play a key role in the development of human sepsis [27]–[29].

**Figure 5:**
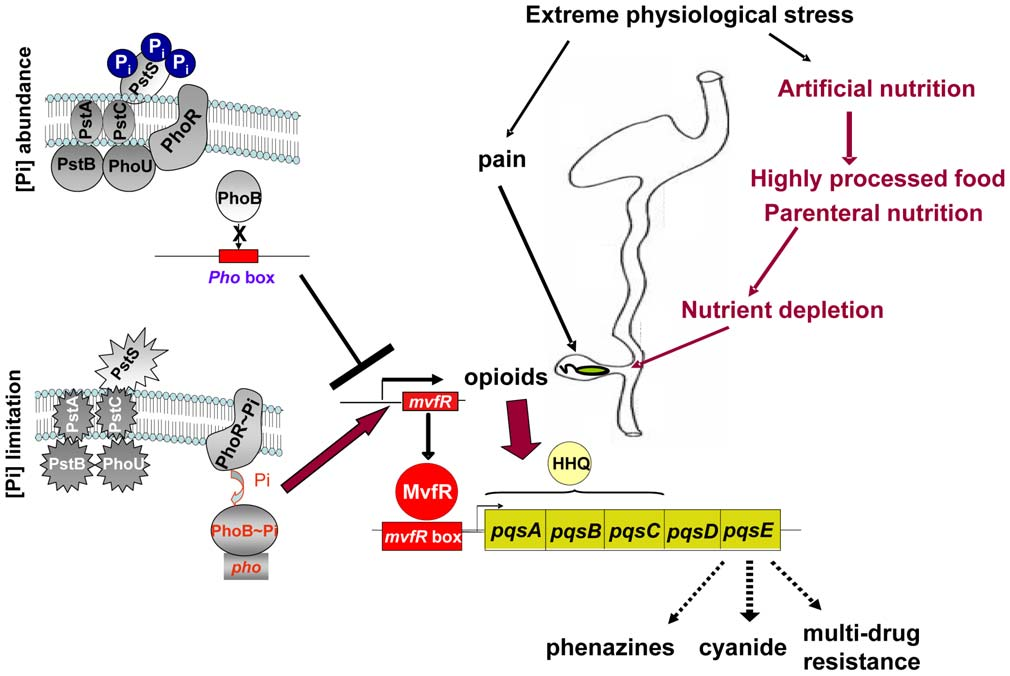
Proposed circumstances under which *P. aeruginosa* is exposed to opioids and phosphate depletion during stress and mechanisms of phosphate-dependent virulence activation or suppression. Critical care treatment destabilizes the indigenous microbiota which becomes replaced by pathogens such as *P. aeruginosa* that colonize the distal gut. Processed foods or parenteral nutrition leads to nutrient limitation in the distal gut where pathogenesis develops. Physiologic stress, pain, and use of analgesics (opioids) coupled with nutrient limitation, promotes premature activation of the MvfR regulated *pqsABCDE* operon leading to the expression of multiple virulent and antibiotic resistant genes mediated by PqsE. When phosphate is abundant expression of these genes is suppressed in *P. aeruginosa*. Mechanisms may include suppression of the PstS-PhoB phosphosensory/phosphoregulatory system that involves phosphate binding to PstS, stabilization of Pst-PhoU-PhoR complex, inability of PhoR to phosphorylate PhoB, and inactivation of the PHO operon including MvfR.

Despite a cell density below that of a “quorum”, the response of *P. aeruginosa* to the k- opioid resulted in high expression of quorum sensing regulated genes and antibiotic resistance efflux pumps. Results suggest that the k- opioid synergizes with HHQ to enhance expression of the *pqsABCDE* operon at low cell density in nutrient poor media and that its expression can be suppressed by extracellular phosphate supplementation (Fig. 5). It has been shown by others that HHQ by itself is not a potent inducer of the transcriptional activation of *pqsABCDE* (100 fold less than PQS) [19]. Transformation of HHQ to PQS, a process that requires LasR, is needed for full activation of the autoregulatory loop of MvfR-PQS. An increase in HHQ alone cannot replace the effect PQS on MvfR as increasing amounts of HHQ negatively affect *pqsA* expression [30]. Under such circumstances, however, based on data from the present study, host stress derived signals (i.e opioids) can provoke earlier and enhanced expression of *pqsABCDE* promoting the biosynthesis of HAQs molecules and PqsE protein. The *pqsABCDE* operon encodes enzymes involved in the biosynthesis of five distinct classes of HAQs [16], [31] including HHQ, a messenger in cell-to-cell communication [16], and 4-hydroxy-2-heptylquinoline N-oxide (HQNO), a potent antibiotic against Gram-positive bacteria [16]. In the present study, the opioid induced expression of *pqsABCDE* that was only 2–3 fold higher compared to its expression without opioids yet this was coupled with suppression of degradation of anthranilate, a precursor of HAQs, suggesting that greater availability for HAQs biosynthesis. The fifth gene of *pqsABCDE* operon, *pqsE*, encodes the PqsE protein that is not required for quinolone biosynthesis yet is under regulation of quinolones, which are ligands of the transcriptional regulator MvfR. Recent studies demonstrate that PqsE functions as a regulator despite lacking a DNA-binding domain and controls the expression of more than 600 genes [30]. Importantly, the regulatory function of PqsE is not dependent on quinolones but is dependent on RhlR [30], [32] that provides the critical link for inter-regulation of MvfR and RhlR. Recently iron was demonstrated to be involved in the PqsE-mediated inter-regulation of MvfR and RhlR [30]. Here we demonstrate, for the first time, that PqsE is involved in opioid- mediated regulation of the core system of quorum sensing in conjunction with phosphate (Fig. 5). Thus, similar to iron, phosphate appears to be primarily involved in the regulation of MvfR expression while opioids are involved in *pqsABCDE* expression. Interestingly, a potential role for PqsE in facilitating environmental adaptation of *P. aeruginosa* to its host has been recently suggested by work from the Williams' laboratory [22] while in the current study we demonstrate that PqsE may play an important role in the adaptive behavior of *P. aeruginosa* during host stress.

An important finding in the present study is the suppression of anthranilate degradation by the k-opioid. Anthranilate is important source for energy metabolism via *antABC* and for quinolone biosynthesis via *pqsA* [33]. That *P. aeruginosa* utilizes anthranilate for either quinolone production or energy may be dictated by distinct conditions including iron availability [34], anthranilate availability [34], or the presence of quorum sensing signals [33], [34]. MvfR has been recently demonstrated to play a critical role in this regard as evidenced by the observation that *antABC* expression is abolished in strains overexpressing MvfR [34]. These studies demonstrate that expression of *pqsA* in MvfR overexpressing strains is highly induced while expression of *antA* is nearly abolished [34]. In view of the data from the present study, *P. aeruginosa* may degrade antharanilate in nutrient poor medium as an internal energy source however the activation of *pqsABCDE* expression induced by the k-opioid will require anthranilate for quinolone production, and thus may prevent its degradation. This suggests a potentially efficient way by which *P. aeruginosa* consumes its own internal energy sources during virulence expression. The observation that phenazine operons were up-regulated by k-opioids may indicate that *P. aeruginosa* uses its own redox-active secondary virulence metabolites as electron acceptors during energy generation. This integration of metabolism and virulence may be involved specifically when nutrients are limited. The additional function of secondary redox active metabolites such as phenazines may be related to the development of multi-drug resistance during exposure to opioids as others have demonstrated that phenazines are involved in the regulation of Mex pumps [35]. Therefore in response to host signals such as opioids, whole virulence networks and resistance to multiple antibiotics in *P. aeruginosa* become activated, which might apply to outbreaks of particularly virulent and difficult to eradicate strains.

The regulatory role of phosphate on bacterial virulence is highly conserved and well established for many pathogens of importance to human health [9], [24], [36]. Work from our laboratory has demonstrated that phosphate concentration signals a life or death response in *P. aeruginosa* against both *C. elegans* and mice during stress [9]. Yet data from the present study suggest that phosphate abundance can override the virulence activating effects of host factors released during stress such as opioids. The finding in the present study that host factors released during stress activate virulence in *P. aeruginosa* at low cell densities when nutrient availability is poor but not when phosphate is abundant, underscores the central role of phosphate sensing. As virulence expression has a substantial cost tradeoff, understanding how pathogens balance the decision to invade based on local resources and host tissue factors could reveal novel mechanisms of microbial pathogenesis during host injury. Sensing of phosphate sufficiency as a proxy for resource availability and favorable host health status may eliminate the cost tradeoff and potential population loss faced by a colonizing pathogen and as such, may function as a regulatory checkpoint. Infection prevention strategies that embed host inaccessible resources at the site of pathogen colonization and virulence activation, such as phosphate, may be considered based on the information herein provided (Fig. 5).

In summary, co- processing of environmental cues by *P. aeruginosa* during stress and nutrient limitation may uncover novel pathways of *in vivo* virulence expression and suggest a dual role for host factor stimuli such as opioids and key nutrients such as phosphate.

## Materials and Methods

### Bacterial strains and culture conditions

*P. aeruginosa* strains MPAO1, the parent strain of transposon insertion mutant library in the University of Washington [37], and NPAO1 (Nottingham PAO1) and their derivatives were used in the experiments. TY (10 g/L tryptone and 5 g/L yeast extract), 0.1xTY (1 g/L tryptone and 0.5 g/L yeast extract+0.1 mM potassium phosphate buffer pH 6.0) and 0.1xTY-Pi25 (1 g/L tryptone and 0.5 g/L yeast extract+25 mM potassium phosphate buffer pH 6.0) media were used. For experiments, *P. aeruginosa* was grown in TY medium overnight, and diluted at a ratio of 1∶100 in 0.1x TY. Potassium phosphate buffer, pH 6.0, was included in the 0.1xTY to a final concentration of 0.1–25 mM when needed. After 2 hrs of growth, the kappa-opioid receptor agonist U-50,488 was added to the bacterial cultures to a final concentration of 50–200 µM. Reporter strains MPAO1/*mvfR::lacZ* [4], [9], [19] and ΔPqsA/*pqsA::luxCDABE* [20] were used to determine the expression of *mvfR* and *pqsABCDE*, respectively. NPAO1 derivative mutants ΔPqsE [38] and ΔPqsH were used in qRT-PCR analysis.

### Transmission electron microscopic analysis (TEM)

Bacteria were collected after 7 hrs of growth in 0.1xTY medium and a drop deposited on a Formvar-coated copper grid for 30 s, then rinsed with TE buffer (10 mM Tris-HCl, 1 mM EDTA, pH 8.0) and stained with a 1% aqueous solution of uranyl acetate as previously described [39]. Samples were examined under 300 KV with a FEI Tecnai F30 electron microscope.

### Pyocyanin assay

Cell cultures of *P. aeruginosa* were collected following 7 hrs of exposure to U-50,488 and centrifuged at 5,000× g, 5 min to remove the pellet. One (1) ml of supernatant was extracted using 500 µl chloroform, re-extracted with 150 µl 0.2 M HCl, and then PCN measured at OD 520 nm. Measurements were normalized to initial cell density measured by absorbance at 600 nm.

### Pyoverdin assay

Pyoverdin was measured in black, clear bottom 96-well plates by fluorescence at 400±10/460±40 excitation/emission, using a 96-well Microplate Plate Reader (Synergy HT, Biotek Inc.). Measurements of relative fluorescence units (RFU) were normalized to cell density measured at 600 nm.

### β-galactosidase assay

After 7 hrs of exposure to U-50,488, PAO1/*mvfR'-lacZ* cells [4], [18] were pelleted by centrifugation at 5,000×g, 5 min, and the pellet was kept at −80°C before processing. The β-galactosidase activity analysis was performed as previously described [4].

### Effect of opioid U-50,488, HHQ, and PQS on *pqsABCDE* expression

The *P. aeruginosa* PAO1 derivative ΔPqsA/*pqsA::luxCDABE* was grown overnight in TY medium, and overnight culture was diluted 1∶100 in 0.1xTY and grown for 2 hrs at 37°C, 200 rpm. U-50,488 (2–200 µM), HHQ (2–200 µM), and PQS (0.4–40 µM) were added when needed. Luminescence was measured dynamically using a 96-well Microplate Plate Reader (Synergy HT, Biotek Inc.). Luminescence was measured hourly and normalized to cell density at 600 nm.

### QRT-PCR and microarray analysis

For RNA isolation, cells of *P. aeruginosa* PAO1 were collected after 5 hrs of exposure to 200 µM U-50,488, and 2 ml RNA Protect Bacteria reagent (Qiagen) was added immediately to the 1 ml of bacterial culture followed by the RNA isolation as previously described [9]. For microarray analysis, *P. aeruginosa* MPAO1, the parent strain of transposon insertion mutant library in the University of Washington [37], was used. The microarray analysis was performed using Affymetrix *P. aeruginosa* GeneChips as previously described [9]. Microarray data were deposited in Gene Expression Omnibus (GEO) database, accession number GSE29946. Microarray data is MIAME compliant, and row data has been deposited in a MIAME compliant GEO database as detailed on the MGED Society website http://www.mged.org/Workgroups/MIAME/miame.html.

For qRT-PCR analysis, the *P. aeruginosa* strain NPAO1 (Nottingham PAO1) and its derivative mutants ΔPqsE and ΔPqsH were used. The cDNA synthesis was performed using 1 µg total RNA with the high capacity RNA-to-cDNA kit (Applied Biosystems), and 1 µl of 1∶50 diluted cDNA was used in qRT-PCR analysis in total reaction mixture of 10 µl containing 5 µl of Platinum SYBR Green qPCR SuperMix-UDG with ROX (Invitrogen) and 0.2 µl of 10 µM each primers. The qPCR was performed using 7900HT Fast Real-Time PCR System (Applied Biosystems). The program for amplification had an initial heat step at 50°C for 2 min, followed by the denaturation step at 95°C for 15 sec, and then followed by 40 cycles of 95°C for 15 s and 60°C for 1 min. The specificity of the reaction was monitored by melt-curve analysis following the real-time program. Gene expression was normalized to PA47480. Fold change was determined using normalized expression in 0.1xTY as 100%. The primers previously used [9] and designed with Primer3 software included:

PA2193 *hcnA*-F 5′CACGATATCCAGCCCCTCT3′,

PA2193 *hcnA*-R 5′CATTGAGCACGTTGAGCAC3′,

PA4599 *mexC*-F 5′ATTTGCGTGCAATAGGAAGG3′,

PA4599 *mexC*-R 5′GGCCTCCTGTCGCTCTTC3′,

PA4205 *mexG*-F 5′AACTCGCTCGAAAGCAACTG3′,

PA4205 *mexG*-R 5′TGGCCTGATAGTCGAACAGC3′,

PA1899 *phzA2*-F 5′GGTTTACCGACAACCTGGAATTGC3′,

PA1899 *phzA2*-R 5′AACAGGCTGTGCCGCTGTAACC3′,

PA1249 *aprA*-F 5′CAGCAGGCCCAGGCCAAGT3′,

PA1249 *aprA*-R 5′CGAAGGTCAGGTCGCCCTGAT3′,

PA4748-F 5′AACAAGCAAGGCGGCATCACA3′,

PA4748-R 5′TGCACGGTACGCATTCCAGTGT3′,

PA4210 *phzA1*-F 5′CAGGGCTATTGCGAGAACCACTACA3′,

PA4210 *phza1*-R 5′CACGCAGTTTCTGTATCGGGTTCA3′,

PA2386 *pvdA*-F 5′CCTTCATCGACCTCAACGACAGCTA3′,

PA2386 *pvdA*-R 5′TCGTTGACGAACGGGCTATCGT3′.

### Protein concentration assay

Protein was measured using the BCA Protein Assay Reagent (Pierce).

### Virulence assay of *P. aeruginosa* PAO1 using *Caenorhabdits elegans*

Wild type *C. elegans* N2 provided by the Caenorhabditis Genetic Center (CGC), University of Minnesota, was used in all experiments. Culturing, cleaning, egg preparation for synchronization, and transferring were performed accordingly to the “Maintains of *C. elegans*” (http://www.wormbook.org/chapters/www_strainmaintain/strainmaintain.html).

For experiments, nematodes were grown on *E. coli* OP50 lawns at 25°C up to L4-young adults stage. Nematodes were transferred onto new non-seeded plain agarized plates (ø 30 mm), and 1 ml of Km solution, 100 µg/ml was poured on plate. In 3 hrs, nematodes were transferred into 1.3 ml of 0.1x TY *P. aeruginosa* culture collected after 7 hrs exposure to 50–200 µM U-50,488 or control *P. aeruginosa* culture that was not exposed to U-50,488. In the experiments with phosphate, potassium phosphate buffer, pH 6.0, was included in the 0.1xTY to a final concentration of 25 mM. Incubation of worms was performed at RT and shaking at 60 rpm. 7 worms per plate, 5 plates per experiments were used in each variant, with several independent experiments performed. Mortality of worms was followed, and nematodes were considered dead if they did not respond to the touch of platinum picker.

### Data analysis

Statistical analysis was performed with Student's *t* test using Sigma plot software, and Kaplan-Maier survival graphs using SPSS software. For microarray analysis, GeneChip Operating Software (GCOS) was used for detection of signal intensities. All signals were scaled according to GCOS default target signal value 500. Invariant set normalization was performed using dchip2006 (Affymetrix). The PM-only model was used to generate gene signal intensities. Dchip was used for identification of differentially expressed genes. Thresholds for selecting significant genes were set at a relative difference >1.2-fold and absolute intensity differences between experimental samples and baseline samples >100 and *t* test *P*<0.05.

## Supporting Information

Table S1 Changes in the expression of genes associated with phosphate signaling and acquisition.
(DOCX)

Table S2 Changes in the expression of genes associated with iron signaling and acquisition.
(DOCX)

Table S3 Changes in the expression of genes associated with quorum sensing.
(DOCX)

Table S4 Changes in the expression of genes associated with multi-drug resistance.
(DOCX)

Table S5 Changes in the expression of genes associated with stress response.
(DOCX)
