# Supplementary material for: Pseudomonas aeruginosa Overrides the Virulence Inducing Effect of Opioids When It Senses an Abundance of Phosphate
Source: PLoS One. 2012 Apr 13;7(4):e34883. doi: 10.1371/journal.pone.0034883 (PMC3325935; doi:10.1371/journal.pone.0034883)
Supplement: Table S2 — Changes in the expression of genes associated with iron signaling and acquisition. (DOCX) [file pone.0034883.s002.docx]

**Table S2**. **Changes in the expression of genes associated with iron signaling and acquisition**
